# Supplementary material for: Multi‐dataset identification of innovative feature genes and molecular mechanisms in keratoconus
Source: J Cell Mol Med. 2024 Sep 19;28(18):e70079. doi: 10.1111/jcmm.70079 (PMC11412914; doi:10.1111/jcmm.70079)
Supplement: Supplementary file 2 — Material S2. [file JCMM-28-e70079-s002.zip › 7.GSEA analysis/6.SNORA74B/KEGG_ARRHYTHMOGENIC_RIGHT_VENTRICULAR_CARDIOMYOPATHY_ARVC.html]

Details for gene set KEGG\_ARRHYTHMOGENIC\_RIGHT\_VENTRICULAR\_CARDIOMYOPATHY\_ARVC[GSEA]

|  || Dataset | SNORA74B.group.cls#h\_versus\_l |
| Phenotype | group.cls#h\_versus\_l |
| Upregulated in class | l |
| GeneSet | KEGG\_ARRHYTHMOGENIC\_RIGHT\_VENTRICULAR\_CARDIOMYOPATHY\_ARVC |
| Enrichment Score (ES) | -0.48543718 |
| Normalized Enrichment Score (NES) | -1.4991281 |
| Nominal p-value | 0.029013539 |
| FDR q-value | 0.3122803 |
| FWER p-Value | 0.776 |
Table: GSEA Results Summary

  

Fig 1: Enrichment plot: KEGG\_ARRHYTHMOGENIC\_RIGHT\_VENTRICULAR\_CARDIOMYOPATHY\_ARVC      
 Profile of the Running ES Score & Positions of GeneSet Members on the Rank Ordered List

  

| SYMBOL | TITLE | RANK IN GENE LIST | RANK METRIC SCORE | RUNNING ES | CORE ENRICHMENT || 1 | CACNB1 | na | 316 | 0.683 | 0.0371 | No |
| 2 | CACNG8 | na | 3573 | 0.275 | -0.0382 | No |
| 3 | CACNG5 | na | 3979 | 0.261 | -0.0323 | No |
| 4 | CACNG7 | na | 4118 | 0.256 | -0.0190 | No |
| 5 | DAG1 | na | 6067 | 0.190 | -0.0623 | No |
| 6 | CACNA1F | na | 6293 | 0.183 | -0.0564 | No |
| 7 | EMD | na | 6823 | 0.167 | -0.0603 | No |
| 8 | ITGA4 | na | 7435 | 0.150 | -0.0678 | No |
| 9 | CACNA2D3 | na | 7638 | 0.145 | -0.0638 | No |
| 10 | ITGB7 | na | 7862 | 0.139 | -0.0608 | No |
| 11 | CACNG1 | na | 9619 | 0.100 | -0.1047 | No |
| 12 | ITGA9 | na | 9643 | 0.100 | -0.0986 | No |
| 13 | ITGB6 | na | 9755 | 0.098 | -0.0952 | No |
| 14 | DSC2 | na | 10067 | 0.091 | -0.0980 | No |
| 15 | CACNB2 | na | 10102 | 0.091 | -0.0929 | No |
| 16 | CACNA1D | na | 10286 | 0.087 | -0.0923 | No |
| 17 | DES | na | 10834 | 0.078 | -0.1028 | No |
| 18 | CACNA1C | na | 10871 | 0.077 | -0.0986 | No |
| 19 | DSP | na | 11369 | 0.069 | -0.1083 | No |
| 20 | ACTB | na | 11421 | 0.068 | -0.1051 | No |
| 21 | ATP2A2 | na | 12349 | 0.055 | -0.1281 | No |
| 22 | ACTN4 | na | 12377 | 0.055 | -0.1252 | No |
| 23 | TCF7L2 | na | 12663 | 0.051 | -0.1300 | No |
| 24 | CACNG2 | na | 15359 | 0.021 | -0.2063 | No |
| 25 | DMD | na | 15565 | 0.019 | -0.2109 | No |
| 26 | CTNNA1 | na | 16086 | 0.014 | -0.2250 | No |
| 27 | LMNA | na | 16308 | 0.011 | -0.2306 | No |
| 28 | ITGB4 | na | 17477 | -0.000 | -0.2643 | No |
| 29 | CTNNB1 | na | 17783 | -0.003 | -0.2729 | No |
| 30 | JUP | na | 17968 | -0.006 | -0.2778 | No |
| 31 | ACTG1 | na | 18268 | -0.009 | -0.2858 | No |
| 32 | CTNNA2 | na | 18831 | -0.015 | -0.3010 | No |
| 33 | ITGA2 | na | 22079 | -0.053 | -0.3911 | No |
| 34 | ITGA11 | na | 22351 | -0.056 | -0.3952 | No |
| 35 | GJA1 | na | 22880 | -0.063 | -0.4061 | No |
| 36 | ITGA3 | na | 23349 | -0.071 | -0.4148 | No |
| 37 | ITGA2B | na | 23836 | -0.078 | -0.4236 | No |
| 38 | LAMA2 | na | 24013 | -0.081 | -0.4232 | No |
| 39 | ITGB5 | na | 24428 | -0.088 | -0.4291 | No |
| 40 | ITGA7 | na | 25192 | -0.102 | -0.4443 | No |
| 41 | CTNNA3 | na | 25470 | -0.107 | -0.4450 | No |
| 42 | ITGAV | na | 26724 | -0.133 | -0.4722 | No |
| 43 | TCF7L1 | na | 27185 | -0.144 | -0.4757 | Yes |
| 44 | ITGB3 | na | 27418 | -0.149 | -0.4723 | Yes |
| 45 | ITGB1 | na | 27543 | -0.152 | -0.4656 | Yes |
| 46 | RYR2 | na | 27560 | -0.153 | -0.4557 | Yes |
| 47 | ITGA6 | na | 27950 | -0.163 | -0.4558 | Yes |
| 48 | CACNB3 | na | 27964 | -0.164 | -0.4451 | Yes |
| 49 | ACTN1 | na | 28221 | -0.171 | -0.4410 | Yes |
| 50 | SGCG | na | 28524 | -0.180 | -0.4375 | Yes |
| 51 | TCF7 | na | 28810 | -0.189 | -0.4329 | Yes |
| 52 | SGCD | na | 29062 | -0.196 | -0.4269 | Yes |
| 53 | CACNG6 | na | 29429 | -0.207 | -0.4234 | Yes |
| 54 | ITGA10 | na | 29479 | -0.209 | -0.4107 | Yes |
| 55 | PKP2 | na | 29762 | -0.217 | -0.4041 | Yes |
| 56 | ACTN2 | na | 30345 | -0.239 | -0.4047 | Yes |
| 57 | DSG2 | na | 30526 | -0.246 | -0.3932 | Yes |
| 58 | SGCA | na | 30676 | -0.252 | -0.3805 | Yes |
| 59 | SGCB | na | 31359 | -0.286 | -0.3808 | Yes |
| 60 | CACNG4 | na | 31720 | -0.308 | -0.3704 | Yes |
| 61 | ITGB8 | na | 32130 | -0.330 | -0.3599 | Yes |
| 62 | CDH2 | na | 32366 | -0.345 | -0.3433 | Yes |
| 63 | CACNA2D4 | na | 32845 | -0.380 | -0.3313 | Yes |
| 64 | CACNA1S | na | 33081 | -0.402 | -0.3109 | Yes |
| 65 | ITGA5 | na | 33190 | -0.413 | -0.2861 | Yes |
| 66 | ACTN3 | na | 33200 | -0.413 | -0.2584 | Yes |
| 67 | SLC8A1 | na | 33496 | -0.449 | -0.2365 | Yes |
| 68 | ITGA8 | na | 33651 | -0.470 | -0.2091 | Yes |
| 69 | CACNG3 | na | 33713 | -0.479 | -0.1784 | Yes |
| 70 | CACNA2D2 | na | 33786 | -0.491 | -0.1473 | Yes |
| 71 | ITGA1 | na | 33862 | -0.506 | -0.1152 | Yes |
| 72 | CACNB4 | na | 33958 | -0.525 | -0.0824 | Yes |
| 73 | LEF1 | na | 34351 | -0.633 | -0.0509 | Yes |
| 74 | CACNA2D1 | na | 34671 | -0.912 | 0.0016 | Yes |
Table: GSEA details [plain text format]

  

Fig 2: KEGG\_ARRHYTHMOGENIC\_RIGHT\_VENTRICULAR\_CARDIOMYOPATHY\_ARVC      
 Blue-Pink O' Gram in the Space of the Analyzed GeneSet

  

Fig 3: KEGG\_ARRHYTHMOGENIC\_RIGHT\_VENTRICULAR\_CARDIOMYOPATHY\_ARVC: Random ES distribution      
 Gene set null distribution of ES for **KEGG\_ARRHYTHMOGENIC\_RIGHT\_VENTRICULAR\_CARDIOMYOPATHY\_ARVC**
